# PrivShieldROS: An Extended Robot Operating System Integrating Ethereum and Interplanetary File System for Enhanced Sensor Data Privacy

**DOI:** 10.3390/s24103241

**Published:** 2024-05-20

**Authors:** Tianhao Wang, Ke Chen, Zhaohua Zheng, Jiahao Guo, Xiying Zhao, Shenhui Zhang

**Affiliations:** 1 School of Cyberspace Security (School of Cryptology), Hainan University, Haikou 570228, China; wangtianhao@hainanu.edu.cn (T.W.); chenk@hainanu.edu.cn (K.C.); zhaoxiying@hainanu.edu.cn (X.Z.); 2 College of Intelligence and Computing, Tianjin University, Tianjin 300134, China; 3 College of International Tourism and Public Administration, Hainan University, Haikou 570228, China; 20223008041@hainanu.edu.cn; 4 School of Cyberspace Security, Shanghai Jiao Tong University, Shanghai 200240, China; freak01716@sjtu.edu.cn

**Keywords:** robot operating system (ROS), security of visual sensor data privacy, interplanetary file system (IPFS), HybridABEnc, blockchain

## Abstract

With the application of robotics in security monitoring, medical care, image analysis, and other high-privacy fields, vision sensor data in robotic operating systems (ROS) faces the challenge of enhancing secure storage and transmission. Recently, it has been proposed that the distributed advantages of blockchain be taken advantage of to improve the security of data in ROS. Still, it has limitations such as high latency and large resource consumption. To address these issues, this paper introduces PrivShieldROS, an extended robotic operating system developed by InterPlanetary File System (IPFS), blockchain, and HybridABEnc to enhance the confidentiality and security of vision sensor data in ROS. The system takes advantage of the decentralized nature of IPFS to enhance data availability and robustness while combining HybridABEnc for fine-grained access control. In addition, it ensures the security and confidentiality of the data distribution mechanism by using blockchain technology to store data content identifiers (CID) persistently. Finally, the effectiveness of this system is verified by three experiments. Compared with the state-of-the-art blockchain-extended ROS, PrivShieldROS shows improvements in key metrics. This paper has been partly submitted to IROS 2024.

## 1. Introduction

With the wide application of robot technology in various scenarios, the data security and privacy protection of the robot operating system (ROS) becomes increasingly important. Here, we pay particular attention to the importance of visual sensor data in ROS, which provides ROS with a wealth of information about its surroundings, including object recognition, navigation, and obstacle avoidance. Especially in the field of high privacy protection needs, such as home services, personal care, security detection, and medical assistance, the secure storage and access control of sensitive information collected by vision sensors have become an urgent problem. However, the existing ROS framework was not designed with sufficient consideration for the security of this data, resulting in the problem of data being easily intercepted or tampered with during transmission and storage.

In exploring solutions to the ROS data security problem, we looked at the AuthROS system proposed by other researchers [1]. The system proposes an innovative approach to enhance the security of data exchange and sharing within ROS systems by integrating Ethereum blockchain technology and the SM algorithm family. AuthROS establishes a secure data-sharing framework for ROS by implementing synchronous service-based RPC communication, subject-based asynchronous data flow messaging, and parameter server-based data storage. While AuthROS has successfully enhanced the security of ROS vision sensor data sharing, it has encountered limitations in addressing more complex privacy protection needs, especially in high-throughput application scenarios where blockchain technology reduces data processing efficiency and increases operational costs. In addition, AuthROS falls short in providing a detailed and flexible user authentication and authorization mechanism, which is essential to ensure that only authorized users can access specific sensitive information.

**Figure 1:**
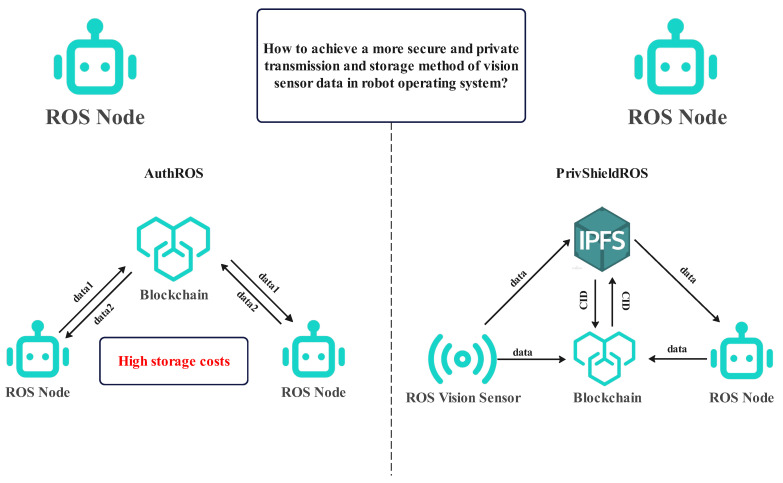
The figure on the left shows the AuthROS framework, which stores data directly on the blockchain with significant resource consumption. Therefore, we adopted IPFS to store the data and store the CID of the data on the blockchain, thus greatly reducing the resource overhead of the blockchain, and the PrivShieldROS framework is shown on the right.

Therefore, we propose a new system scheme to solve the problem of data security storage and access control of ROS vision sensor data in highly privacy-protected scenarios. Our proposed system integrates blockchain technology, Interplanetary File System (IPFS), and attribute-based hybrid encryption (HybridABEnc), and specifically considers the characteristics of vision sensor data. This approach aims to strengthen the security and privacy of vision sensor data storage, ensuring that only authorized users can access specific sensitive information, thus effectively addressing the challenges that ROS currently faces in a high-privacy environment. With this integrated approach, we can better protect the sensitive data collected by vision sensors in high-privacy environments while maintaining the efficiency and operability of the system. The comparison of PrivShielROS and AuthROS frameworks is shown in Figure 1.

In this system, the application of blockchain technology not only ensures the immutability and transparency of the private data in the vision sensor but also greatly reduces the risk of data being illegally modified or leaked. This risk reduction is due to the decentralized nature of the blockchain, in which data is not stored on a single server or location but is dispersed across multiple nodes of the network. Therefore, even if part of the node is damaged or fails, the rest of the system can still operate normally, thus ensuring the security and integrity of the vision sensor data. In addition, our solution utilizes the distributed network architecture of IPFS to provide a durable and efficient storage solution for data generated by vision sensors. IPFS is a distributed file storage system that allows users to efficiently store and access files, applications, and data in a decentralized manner. By decentralizing data storage across nodes worldwide, IPFS effectively overcomes the single point of failure and scalability limitations faced by traditional centralized storage systems. In IPFS, each piece of data is identified by a unique hash value, ensuring efficient and accurate retrieval and access even if the data is distributed across different network nodes.

In addition, in order to achieve accurate control and protection of sensitive vision sensor data, we also introduce key policy-based attribute encryption (KP-ABE) technology, which enables system administrators to establish access control policies according to user attributes so as to achieve accurate control of sensitive data. In this system, data privacy protection and access control no longer rely on the traditional user name and password authentication mechanism but develop attribute-based access control. This approach provides greater flexibility and security because even if the user’s credentials are compromised, individuals who do not meet the attribute requirements will still be unable to access the encrypted vision sensor’s private data information. This ensures that only users who meet certain attribute conditions can decrypt and access the encrypted data content, thus enhancing the security and flexibility of private data access. To further improve the security and processing efficiency of the system, we have adopted a hybrid attribute encryption scheme that combines KP-ABE and Advanced Encryption Standard (AES). In this scheme, KP-ABE is specifically designed to encrypt symmetric keys critical to data access, while AES is used to encrypt the data itself. As AES is an efficient symmetric encryption algorithm, this hybrid encryption method ensures the security of the system and greatly improves its encryption efficiency. This strategy not only enhances data security but also simplifies the encryption process. In this hybrid model, even if the KP-ABE encryption attribute key is partially compromised, an unauthorized user cannot obtain the AES key and, therefore, cannot decrypt the stored or transmitted vision sensor privacy data. Furthermore, even if an attacker gains access to some encrypted data, they still cannot access the actual content without the proper KP-ABE attributes, thus ensuring the confidentiality and integrity of the data. The architecture provides robust and flexible data protection mechanisms that transcend the limitations of traditional security models and adapt to today’s dynamic and highly fragmented digital environment.

The advantages of our system are mainly reflected in the following aspects: it provides a safe, efficient, and reliable storage solution for the private data of vision sensors generated in the ROS environment; it ensures data immutability and transparency through the recording of the CID on the blockchain; and the multilevel security protection mechanism integrating AES and KP-ABE provides strict security protection and access control for highly private data in ROS vision sensors.

The contributions can be summarized as follows:

(1) We propose a new framework and further develop a blockchain-extended robot operating system that leverages distributed storage technologies (IPFS), enhanced attribute-based encryption for fine-grained access control of private data from visual sensors, and distributed authentication mechanisms for secure user authentication. Enhanced security for user authentication ensures the security and privacy of data collected by robotic vision sensors when stored and accessed.
(2) By combining blockchain technology with the Interplanetary File System (IPFS), leveraging the content-adding mechanism of IPFS and the immutability of blockchain, we provide a more secure and reliable storage solution for sensitive vision sensor data in ROS environments.
(3) By integrating KP-ABE with AES, the attribute-based HybridABEnc mechanism not only achieves fine-grained access control of vision sensor privacy data, ensuring that only users meeting specific attributes can decrypt and access the encrypted vision sensor data, but also enhances the encryption efficiency for processing vision sensor data [2]. This significantly improves the security of private data, ensuring its protection even in complex ROS application scenarios.

The rest of this paper is organized as follows: Section 2 reviews relevant studies; Section 3 formalizes our approach and describes our architectural design in detail; Section 4 analyzes the experimental results thus demonstrating the feasibility and benefits of the mechanism. The paper concludes with a summary of these findings.

## 2. Related Work

In this section, we review the work in the literature on blockchain, the distributed data system IPFS, and the application of attribute encryption in robotic operating systems (ROS). These studies form the theoretical and technical basis for our proposed system.

Recent research demonstrates diverse advances and applications in robotic operating systems (ROS) and blockchain technology convergence. Zhang et al. bridge ROS and the Ethereum blockchain through the Ros-Ethereum tool, providing AuthROS, a new solution for secure data sharing among robots. This solution uses smart contracts and SM encryption technology to ensure the security of data transmission and the immutable nature of private data flows [1,3]. Mallikarachchi et al. implemented the collaborative task management of different types of robots through the Swarm Contracts smart contract framework, marking the first practical application of ROS integration with the Ethereum blockchain while maintaining interoperability and trust [4]. Roy et al. combined cloud computing and blockchain technologies to use UAVs and unmanned ground vehicles to evaluate unmanned aerial vehicles’ safe coordination and autonomous surveillance capabilities, highlighting the importance of multi-robot coordination and modular robots [5]. Similarly, Salimi et al. have proposed a multi-robot system framework built on the Hyperledger Fabric blockchain platform. By implementing blockchain authentication and smart contracts among robots, they ensure the security and efficiency of the collaborative process while also exploring the potential and importance of applying blockchain technology in industrial environments [6]. Zhang et al. review current innovative architectures and technologies applicable to robotic systems and discuss existing technical challenges and preparations for advancing distributed robotic systems [7]. Salimi et al. describe the integration of ROS 2 with the Hyperledger Fabric blockchain, emphasizing the importance of enabling secure data aggregation and control in autonomous robotic systems [8]. Morón et al. integrated blockchain technology with the UWB positioning system to improve the security and credibility of multi-robot systems [9]. In the study by Fu et al., the application of Hyperledger Fabric blockchain technology for remotely operated mobile robots was explored, with a particular focus on integrating this technology with ROS 2 systems [10]. Through this integration, the research demonstrates the potential of blockchain technology to improve the security and auditability of mobile robot operations. Keramat et al. demonstrated the integration of ROS 2 with IOTA’s smart contract platform. This novel integration provides a more scalable and partition-tolerant DLT solution for multi-robot systems with intermittent connectivity [11]. These studies not only extend the application of ROS in high-level decision-making and secure communication but also provide new solutions for trust and security in distributed robot systems, demonstrating the wide application and potential of blockchain technology in robot systems.

However, existing blockchain technology may limit the performance and scalability of multi-robot systems due to its inherent high latency and resource consumption during implementation. To this end, we combine blockchain with IPFS to optimize multi-robot collaboration systems. In application, IPFS can improve performance and provide better content services. For example, Y. Chen et al. proposed research on optimizing the IPFS structure through a triple copy and erasure code storage scheme [12]. Onwubiko et al. proposed a blockchain-based solution to the digital twin’s information security and trust challenges. The solution integrates smart contracts and IPFS to enhance asset data’s security and transmission efficiency [13]. In addition, Sonkamble et al. have introduced a new type of healthcare data management architecture based on blockchain and IPFS. By applying rigorous algorithms and smart contracts, they ensure secure storage, access control, and privacy protection of electronic health records (EHRs) [14]. In a development related to secure file sharing, M. Steichen et al. introduced aclIPFS, a system that combines IPFS and Ethereum smart contracts. It uses Ethereum smart contracts to manage access rights to achieve secure access rights management [15]. In the field of auto insurance claims, Chen et al. designed a new auto insurance claims system by integrating blockchain and IPFS technology. They used encryption technology such as ECDSA to ensure the security and integrity of data, successfully solving the challenges of insurance fraud and data traceability and reducing storage costs [16]. Similarly, Sangeeta et al. applied blockchain and IPFS to road safety monitoring and significantly improved the security, data integrity, and economic efficiency of monitoring data by persisting data hash value storage and adding a keyword search function [17]. Additionally, Y. Chen and M. Steichen’s research combines blockchain with IPFS to improve storage performance and interactivity. N. Nizamuddin et al. proposed a blockchain-based solution for document sharing and version control, enabling multi-user collaboration and tracking changes [18]. However, there is no explicit mention of encryption protections for document content or how to manage and control user access to documents. H. Hasan et al. ensured the originality and authorship of online books and documents by combining IPFS and Ethereum smart contracts [19]. In addition, R. Kumar et al. designed a blockchain storage model based on IPFS to solve the efficiency problem of storing and accessing transactions in the blockchain network [20]. The research work of these scholars has realized solutions for specific application scenarios based on blockchain and IPFS. Still, there is no particular emphasis on the security of the data itself, and there is no good solution for controlling access rights.

Although existing research provides architectures for implementing specific functions on blockchain and IPFS, most studies have yet to emphasize the security of the data itself and the effective enforcement of permission controls. Given this, the fusion application of attribute encryption becomes the focus of our consideration. For example, X. Chen et al. proposed an attribute-based anti-quantum signature technology (AQ-ABS) aimed at anti-quantum computing attacks, which is used for secure sharing of electronic medical records (EMR) and combined with blockchain [21]. By analyzing decentralized storage and data encryption, Van-Duy Pham et al. built a storage system that integrated the IPFS network, attribute-based encryption (ABE), and MA-ABE encryption technology and realized the secure storage of data by integrating Ethereum blockchain [22]. However, their approach does not specifically explain how to revoke or modify access for specific users or entities to address changing requirements. To address these evolving data security and privacy needs, Kallapu et al. have developed a new, blockchain-based, attribute-aware encryption approach. This method combines attribute encryption technology and access control policies designed specifically for cloud data, thus significantly improving the security level of data processing and privacy protection [23]. Tao et al. proposed a medical file-sharing scheme that combines blockchain and decentralized attribute encryption technology to achieve fine-grained access control and ensure data privacy and security [24]. Aiming at the low efficiency and inflexibility of traditional attribute encryption, Guo et al. introduced an efficient, traceable, and dynamic access control attribute encryption scheme (table-DAC), which is supported by blockchain technology and achieves more reliable data access management [25]. At the same time, X. Lu et al. introduced a fine-grained access control scheme integrated with CP-ABE, designed specifically for data sharing among IoT devices, and ensured the security and confidentiality of the information exchange process [26]. In addition, they propose a fine-grained access control mechanism that combines symmetric encryption and CP-ABE to ensure the security of IoT data and the effective control of data owners over visitors. Regarding privacy protection, H. D. Hoang et al. proposed a scheme called BABEHealth, which uses blockchain and CP-ABE technology to establish true ownership of electronic medical records for patients [27]. However, CP-ABE, as utilized in these studies, exhibits limitations regarding access control accuracy for data owners compared to KP-ABE. This is due to the direct association of the policy with the ciphertext, which makes it challenging to manage data access flexibly.

Therefore, based on the synthesis of these research findings, our scheme highlights the utilization of KP-ABE attribute encryption and the integration of the AES algorithm. Through this dual-layer encryption and access control mechanism designed for vision sensor data, we offer more secure and manageable private data storage solutions for various robotic operating environments, particularly those with high privacy requirements such as home automation, safety monitoring, and medical care. The KP-ABE algorithm enables precise control by data owners over who can access specific data through defining access policies, while the combined AES algorithm is employed to encrypt the actual data content. Both measures reduce the risk of key exposure and establish a robust security foundation for private information stored in ROS.

## 3. Materials and Methods

In our system architecture, the initial steps begin with a user interface. This interface provides an efficient platform for human-computer interaction, enabling users to monitor and manage the communication process between automated nodes in real-time. The system adopts the publish/subscribe messaging model, effectively delivering theme-driven messages across different nodes. Additionally, it boasts excellent data processing and storage capabilities, allowing it to receive, process, and stably store data. The system can collect multi-modal sensing data, such as environmental images, and securely store this data on local storage devices to ensure high data security and reliability.

In the data processing stage, the system applies a hash algorithm to each collected data sample, generating a unique and securely encrypted hash value. This hash value is designed to ensure the data’s uniqueness, persistence, and immutability. These generated hashes are then uploaded to the blockchain via the integrated messaging middleware within the system, creating a permanent, immutable check code for each data piece, thus providing additional assurance of the data’s immutability and continuous verifiability.

Regarding data confidentiality, the system employs attribute-based hybrid encryption (HybridABEnc) to encrypt privacy data from vision sensors. This encryption mechanism facilitates precise access control by allowing only users who meet specific attribute criteria to access and decrypt the designated data, thereby significantly enhancing protection against unauthorized access. The encrypted data is then uploaded to a distributed storage solution and synchronized to the IPFS network through local IPFS nodes. IPFS employs content addressing to ensure data consistency and resistance to tampering through content identifiers (CID).

**Figure 2:**
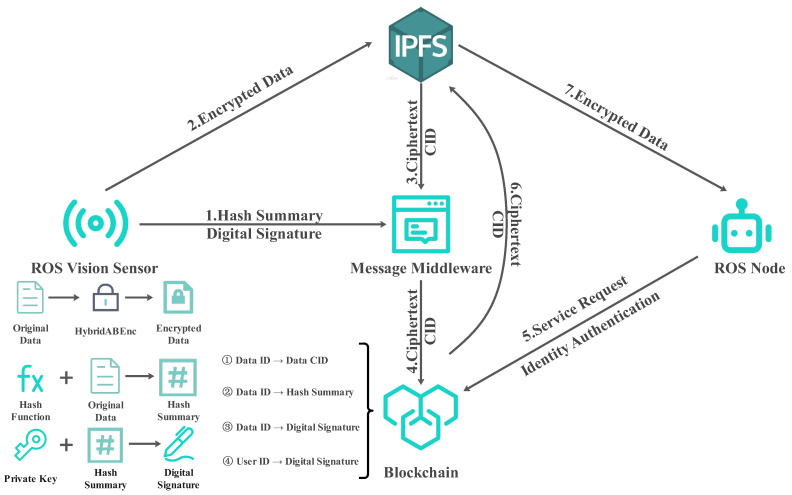
The framework of the PrivShieldROS. The system includes two parts: data processing and storage, and data acquisition and verification. Processes 1, 2, 3, and 4 involve the processing and storage of data; Processes 5, 6, and 7 are responsible for data acquisition and related validation operations. In addition, we store the mappings on the blockchain, labeled ➀, ➁, ➂, and ➃, to support the functionality of the system. The message middleware in the figure is a component that is used to process and deliver messages. In the distributed system, the message middleware acts as the bridge of message passing, which enables different components to communicate with each other asynchronously. The experimental part of rabbitMQ is a kind of message middleware.

Finally, the CID value of vision sensor privacy data is recorded on the blockchain, providing unequivocal evidence of the data’s originality and traceability. Hence, based on the CID value, the state of the data at any given time can be independently verified, ensuring that the data remains unchanged since its upload. This process ensures high levels of security, trust, and reliability throughout the data lifecycle, encompassing storage, sharing, and utilization. The framework of the system is illustrated in Figure 2.

### 3.1. Security Mechanism to Ensure Data Privacy and Integrity of ROS Nodes

In the ROS node, we introduce a symmetric encryption mechanism (AES), a key policy-based attribute encryption mechanism (KP-ABE), a hash digest, and digital signature technology. The Advanced Encryption Standard (AES) is a symmetric key encryption algorithm commonly used to quickly and securely encrypt privacy data collected from vision sensors. Key-Policy Attribute-Based Encryption (KP-ABE) is a form of attribute-based encryption that associates access control policies with keys to implement fine-grained access control. In KP-ABE, users possess a specific set of attributes and the corresponding secret attribute key. The key is generated according to access control policies, which are expressed as Boolean formulas based on attributes. This approach ensures that only users whose attributes satisfy the access control policy can decrypt the ciphertext using their secret attribute key. The encryption mechanism based on key policy attributes is a flexible tool that can adapt to various data access control requirements. The encryption and decryption processes are detailed in Algorithms 1 and 2.

#### 3.1.1. Granular Access Control Based KP-ABE

In PrivShieldROS, A represents the access control structure, M is the message, γ is a set of attributes, PK are the public parameters, MK is the master key, E is the ciphertext, and D is the decryption key. From the perspective of the framework’s users, it can be divided into Initialization, Encryption, Key Generation, and Decryption.

**Figure 3:**
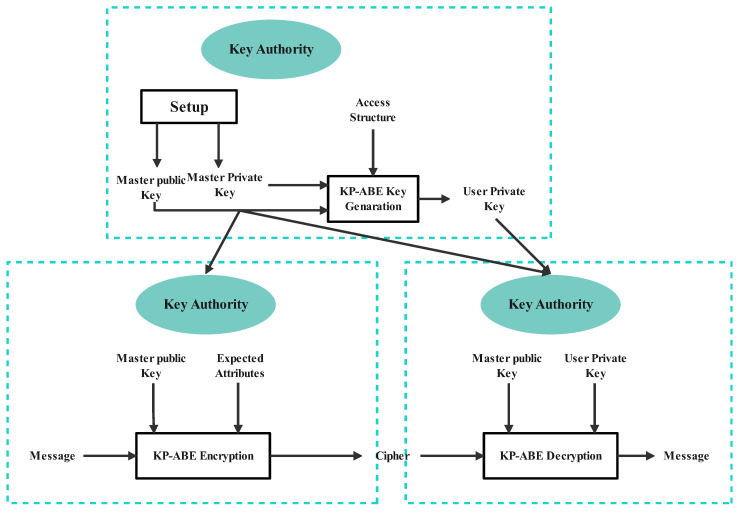
Schematic diagram of KP-ABE algorithm.

The following is the specific algorithm process:

Access tree T. Let T be the tree representing the access structure. Each non-leaf node of the tree represents a threshold gate, described by its children and a threshold value. If ${\mathrm{num}}_{x}$ is the number of children of node x and $k_{x}$ is the threshold of node x, $0<k_{x}\leq {\mathrm{num}}_{x}$. The threshold is an OR gate when $k_{x}=1$. When $k_{x}$ = ${\mathrm{num}}_{x}$, the threshold gate is an AND gate. Each leaf node x of the tree is described by the attribute and the threshold $k_{x}$ = 1. The schematic diagram of the KP-ABE algorithm is shown in Figure 3.

To facilitate the use of access trees, we define some functions. We use parent(x) to represent the parent of node x in the tree. The function att(x) is defined only if x is a leaf node and represents the properties associated with the leaf node x in the tree. The access tree T also establishes the hierarchy among the children of each node, wherein the children are sequentially numbered from 1 to num. The function index(x) returns the number associated with node x. Where the index value is uniquely assigned to a node in the access structure of a given key in any manner.
**Algorithm 1** Attribute-Based Encryption: Data Encryption Procedure1:**procedure**EncryptData2:     data_path←input(‘‘Enterthedatapathtoencrypt’’)3:     access_policy←input(‘‘Entertheattributelist’’)4:     **print** (access_policy)5:     access_key←input(‘‘Entertheaccesscontrolpolicy’’)6:     **print**(access_key)7:     pk_deserialized←Deserialize(‘pk_serialized.pkl’)8:     mk_deserialized←Deserialize(‘mk_serialized.pkl’)9:     sk←KeyGen(pk,mk,access_key)10:  SerializeAndSave(sk,‘sk_data’)11:  data←Readdata(data_path)12:  encrypted_data←Encrypt(pk_deserialized,data,access_policy)13:  SerializeAndSave(encrypted_data,‘encrypted_data’)14:  **print** (“Encryption complete, encrypted data path”)15:  DeleteData(data_path)16:  **print** (“Original data deleted”)17:**end procedure**

**Algorithm 2** Attribute-Based Encryption: Data Decryption Procedure
1:
**procedure** DecryptData
2:     encrypted_data_path←input(‘‘Enterthedatapathtodecrypt’’)3:     encrypted_data←Deserialize(‘encrypted_data’)4:     sk←Deserialize(‘sk_data’)5:     decrypted_data←Decrypt(encrypted_data,sk)6:     SaveToData(decrypted_data,‘decrypted_data’)7:     **print** (“Decryption complete, decrypted data path: ‘decrypted_data’”)8:
**end procedure**



Let $G_{1}$ be the bilinear group of prime order ’p’ and ’g’ be the generator of $G_{1}$. In addition, let $e:\;G_{1}\times G_{1}\to G_{2}$ represent bilinear mappings. The safety parameter κ determines the size of the group. At the same time, define the Lagrange coefficient $\Delta_{i,S}\;\mathrm{for}\;i\in Z_{p}$ and the set S of elements in $Z_{p}$:$\Delta_{i,S}\left(x\right)=\prod_{j\in S,j\neq i}\frac{x-j}{i-j}$. Associate each attribute with a unique element in $Z_{p}^{*}$.

(1) Initialization. Define the property field U={1,2,…,n}. Now, for each attribute i∈U, a number $t_{i}$ is selected uniformly at random from $Z_{p}$. Finally, y is uniformly randomly selected in $Z_{p}$. The published public argument PK is $T_{1}=g^{t_{1}},\ldots ,T_{\left|u\right|}=g^{t_{\left|u\right|}},Y=e{\left(g,g\right)}^{y}$. The master key MK is as follows:
  (1) $$t_{1},\ldots ,t_{\left|u\right|},y$$
(2) Encryption (M,γ,PK). To encrypt the message $M\in G_{2}$ under a set of attributes γ, select a random value $s\in Z_{p}$ and publish the ciphertext as follows:
  (2) $$E=(\gamma ,E^{\prime }={\mathrm{MY}}^{s},{\left\{E_{i}=T_{i}^{s}\right\}}_{i\in }\gamma )$$
(3) Key generation (T,MK). The algorithm produces a key that allows the user to decrypt messages encrypted under a set of attributes γ only if T(γ)=1. First select the polynomial $q_{x}$ for each node x (including the leaves) in the tree T. These polynomials are selected from the root node r in a top-down manner in the following way.

For each node x in the tree, set the degree $d_{x}$ of the polynomial $q_{x}$ to be one less than the threshold $k_{x}$ for that node, namely the $d_{x}=k_{x}-1$. Now, for the root node r, set $q_{r}\left(0\right)=y$ and randomly set the other points $d_{r}$ of the polynomial $q_{r}$ to fully define it. For any other node x, set $q_{x}\left(0\right)=q_{\mathrm{parent}(x)}\left(\mathrm{index}\left(x\right)\right)$ and select $d_{x}$ other points at random to fully define $q_{x}$. Once the polynomial is determined, for each leaf node x, the user obtains the following secret values:

(3) $$D_{x}=g^{\frac{q_{x}\left(0\right)}{t_{i}}}\mathrm{where}\;i=\mathrm{att}\left(x\right)$$

The set of the above secret values constitutes the decryption key D.

(4) Decryption (E,D). The decryption process is specified as a recursive algorithm. First, a recursive algorithm DecryptNode(E,D,x) is defined. This algorithm takes as input the ciphertext $E=(\gamma ,E^{\prime },{\left\{E_{i}\right\}}_{i\in \gamma })$, the private key D (assuming the access tree T is embedded in the private key), and the node x in the tree. It outputs group elements of $G_{2}$ or ⊥. Let $i\;=\;\mathrm{att}\left(x\right)$. If node x is a leaf node, then
  (4) $$\mathrm{DecryptNode}\left(E,D,x\right)=\left\{\begin{array}{c} e\left(D_{x},E_{i}\right)=e\left(g^{\frac{q_{x}\left(0\right)}{t_{i}}},g^{s\cdot t_{i}}\right)=e{\left(g,g\right)}^{s\cdot q_{x}\left(0\right)},\;\mathrm{if}\;i\in \gamma \\ ⊥,\;\mathrm{otherwise} \end{array}\right.$$

Let us discuss the scenario where x is a non-leaf node in the recursion case. For all x child nodes z, it calls DecryptNode and stores the output as $F_{z}$. Let $S_{x}$ be the set of children z of any $k_{x}$ size such that $F_{z}\neq ⊥$. If no such set exists, then the node is not satisfied and the function returns ⊥. Otherwise, the following calculations should be performed and the results returned:

(5) $$\begin{array}{cc} F_{x} & =\prod_{z\in S_{x}}F_{z}^{\Delta_{i.S_{x}^{\prime }}\left(0\right)},\;{\mathrm{where}}_{S_{x}^{\prime }=\left\{\mathrm{index}\left(z\right):z\in S_{x}\right\}}^{i=\mathrm{index}(z)}\\ \; & =\prod_{z\in S_{X}}{\left(e{\left(g,g\right)}^{s\cdot q_{z}\left(0\right)}\right)}^{\Delta_{i,s_{x}^{\prime }}\left(0\right)}\\ \; & =\prod_{z\in S_{x}}{\left(e{\left(g,g\right)}^{s\cdot q_{\mathrm{parent}(z)}\left(\mathrm{index}\left(z\right)\right)}\right)}^{\Delta_{i,s_{x}^{\prime }}\left(0\right)}\;\left(\mathrm{by}\;\mathrm{construction}\right)\\ \; & =\prod_{z\in S_{x}}e{\left(g,g\right)}^{s\cdot q_{x}\left(i\right)\cdot \Delta_{i,s_{x}^{\prime }}\left(0\right)}\\ \; & =e{\left(g,g\right)}^{s\cdot q_{x}\left(0\right)}\;\left(\mathrm{using}\;\mathrm{polynomial}\;\mathrm{interpolation}\right) \end{array}$$

Now that the function DecryptNode has been defined, the decryption algorithm just needs to call the function at the root of the tree. If and only if the ciphertext satisfies the tree, $\mathrm{DecryptNode}\left(E,D,r\right)=e{\left(g,g\right)}^{\mathrm{ys}}=Y^{s}$. Because $E^{\prime }={\mathrm{MY}}^{s}$, the decryption algorithm simply splits out $Y^{s}$ and recovers the message M.

#### 3.1.2. The Integrity and Credibility of the Data

Although HybridABEnc provides the flexibility to encrypt and decrypt privacy data collected from vision sensors according to access control policies, its mechanism is confined to the encryption and decryption processes and does not directly provide a means to verify the integrity and authenticity of the data. We introduce a hash digest as a validation mechanism to address this limitation. After the original data is encrypted with HybridABEnc, we hash the original data and store the resultant hash value on the blockchain as proof of integrity. The immutable property of the blockchain adds an extra layer of assurance for the authenticity of the hash value. After decryption, we can recalculate the hash value and compare it to the hash value stored within the ciphertext, thereby ensuring the integrity of the original data. Furthermore, we incorporate digital signatures to confirm the sender’s identity and enhance the system’s security. Digital signatures are commonly used to verify the identity of the sender of data. They are created by encrypting the summary hash of the data with the data owner’s private key. This secures not only the integrity and authenticity of the information but also confirms the sender’s identity and ensures the non-repudiation of the data sender.

#### 3.1.3. RBE Exchange Framework (ROS-Ethereum)

We have utilized an exchange framework called ROS-Ethereum to bridge ROS and the blockchain for potential security vulnerabilities in the interaction between ROS and the blockchain. ROS does not inherently provide the capability to interact directly with the blockchain. Therefore, through RBE, we have endowed ROS with the ability to interface with the Ethereum blockchain. RBE employs UDP communication mechanisms, integrates Ethereum, utilizes the Web3 framework, and incorporates the SM algorithm family to provide secure and reliable communication capabilities. Furthermore, the framework allows users to monitor node activity, preprocess messages, and interact with Ethereum through the Web3 interface, ensuring the reliability and security of data exchange. With RBE, users can effortlessly invoke smart contracts on Ethereum to facilitate interactions with the blockchain.

**Figure 4:**
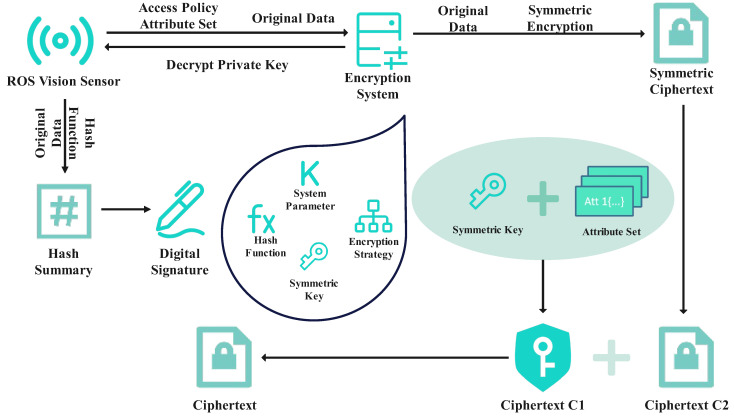
ROS node part flow chart.

In the ROS node, the system administrator initially generates and discloses system parameters during the initialization phase. This includes security parameters and the algorithm parameters used in the encryption mechanisms. Secondly, each user creates a private key based on their unique attributes. The private key generation algorithm leverages the user’s attributes and the system’s algorithm parameters to produce the corresponding private key. Subsequently, the system randomly generates a symmetric key and uses the symmetric encryption algorithm (AES), along with the key, to encrypt the privacy data collected from vision sensors, thereby generating the symmetric ciphertext. Before encryption, the ROS node hashes a summary of the original data to verify its authenticity and integrity. The system encrypts the symmetric key in policy encryption according to the attribute set. This attribute set can be composed of attributes such as the user’s age, job title, organization, etc. Finally, the system encapsulates the encrypted symmetric ciphertext and encrypted symmetric key into a complete ciphertext packet. Therefore, the final policy ciphertext consists of the plaintext after encryption and the symmetric key after encryption. The execution process is illustrated in Figure 4.

### 3.2. Blockchain Smart Contracts Integrate with ROS

**Figure 5:**
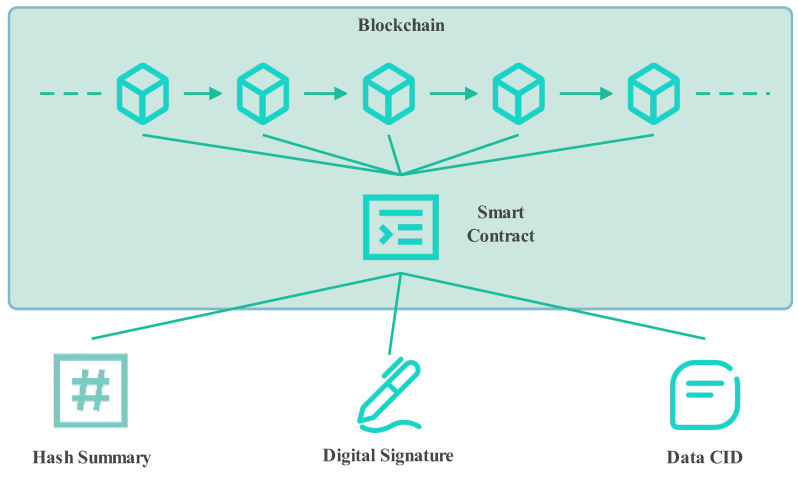
Smart contract part diagram.

The integrity and immutability of encrypted files rely on blockchain technology, which establishes a decentralized distributed ledger shared among all network participants based on a consensus mechanism. This design eliminates the need for third-party validators, making the overall system more secure and decentralized. In the ROS-integrated blockchain system, smart contracts play a central role. They are self-executing contracts deployed on the blockchain that can operate independently on the network nodes. These contracts are programmed to self-execute and validate preset conditions and terms automatically. The associated smart contract is activated when a ROS-based application initiates a transaction to an Ethereum address and provides the corresponding input parameters. Ethereum is an open-source, distributed computing platform that supports the development of decentralized applications (DApps) through its robust smart contract capabilities. It enables ROS developers to write code directly on the blockchain, allowing for the customization of decentralized applications for robots or automated systems. Each node in the Ethereum network possesses a unique Ethereum address (EA) for interactions. In this architecture, the Ethereum blockchain serves as an immutable data repository and the execution layer for smart contracts, while the ROS system manages routine robot control tasks, such as processing sensor data, making decisions, and executing control commands. The smart contract component of the system is illustrated in Figure 5.

#### 3.2.1. Consensus Mechanism

Consensus algorithms play a crucial role in ensuring the immutability, automation, and support for anonymous transactions of blockchain-distributed ledgers. Without these algorithms, blockchains would be reduced to inefficiently distributed ledgers. Consensus algorithms are fundamental to the core meaning and value of blockchain technology. Common consensus algorithms currently include Proof of Work (PoW), Proof of Authority (PoA), Proof of Stake (PoS), and Practical Byzantine Fault Tolerance (PBFT). Ethereum is presently utilizing the PoW consensus algorithm, but with the upcoming launch of ETH2.0, it is poised to transition to the PoS algorithm. The most significant difference between PoW and PoS lies in their validation methods for blocks. PoW requires mining operations to validate blocks, which does not guarantee block time, while PoS can achieve a more predictable block time without the need for mining operations. On the other hand, PoA validates blocks through pre-authorized nodes without involving mining, facilitating predetermined block times.

The consensus algorithm aims to solve the problem of maintaining data consistency among nodes in a distributed system. According to CAP theory, two of the three requirements can be satisfied at most: consistency, availability, and partition fault tolerance. Therefore, the choice of consensus algorithm must take into account the specific requirements of practical application scenarios, as well as the uniqueness of different types of blockchains. The PoW algorithm obtains the accounting right by competing for computing power, which ensures that the blockchain network security is mainly mastered on the nodes with large computing power. This mechanism improves the decentralization and security of the network to a large extent but at the corresponding cost of sacrificing a certain transaction processing efficiency. However, for application scenarios with high privacy requirements, ensuring the system’s security is far more important than pursuing the ultimate efficiency. In contrast, the PoS algorithm determines the accounting right through the proof of stake, which greatly improves the system’s efficiency but may introduce security risks due to the concentration of wealth. On the other hand, the PBFT algorithm can ensure a certain percentage of fault tolerance and performs better than PoW. However, its high communication cost limits the scalability of the system.

Considering that our system is oriented to large-scale application scenarios with high requirements for privacy and security, in most cases it does not have extremely strict requirements for real-time transactions. The PoW consensus algorithm is obviously a more appropriate choice. Although a part of the efficiency is sacrificed, it greatly improves the ability of the system to resist attacks and malicious behaviors. It ensures the stable operation of the system and the security of user privacy information. Therefore, the adoption of the PoW algorithm is appropriate for our system.

#### 3.2.2. Interactive Program

Interaction mechanisms: The blockchain end automatically retrieves messages for processing and interaction. Interactions with Ethereum are primarily facilitated through smart contracts developed using Solidity, a programming language specifically designed for Ethereum contract development. On the Ethereum platform, after obtaining the CID value of encrypted data, one employs Solidity to write smart contracts and define structures for storing data details, such as ‘data ID: data CID’, ‘data ID: data hash summary’, etc. The functions of these contracts encompass adding and querying data. Upon composing the contract, we utilize Ethereum’s tool, Remix, to compile and deploy the contract, acquiring the contract address after deployment. We use Web3.js to connect to the Ethereum network via the client, employing the contract’s Application Binary Interface (ABI) and address to instantiate the contract object. We then invoke the contract function to execute a transaction. Permission control settings ensure only the contract creator or data owner can update data information. After the transaction is dispatched, it is monitored until confirmation; upon confirmation, the CID value of the encrypted data is recorded on the Ethereum blockchain. The relevant algorithms are described in detail in Algorithm 3. The gas consumption of smart contract deployment is shown in Table 1. The table illustrates the deployment process of the smart contract and the gas consumption required for three ’store’ function calls. The differences in gas consumption between the three functions are attributed to the amount and type of data they process and store and the complexity of the operations involved in processing these data. The storeDataIdToHashAndSignature function is the most complex processing data, performs the most complicated operations, and leads to the highest gas consumption. The storeDataIdToCid function handles the simplest data and uses the least gas. The storeUserIdToSignature function is in the middle regarding data complexity and gas consumption. Additionally, three ’read’ functions in the smart contract solely access local data and thus do not incur any gas consumption. The generation of the hash function uses the SHA256 algorithm.
**Algorithm 3** Smart Contract Algorithm Pseudo-code1: **mapping** (uint256 ⇒ string) dataIdToCid2: **mapping** (uint256 ⇒ string) dataIdToHashAndSignature3: **mapping** (uint256 ⇒ string) userIdToSignature4: **function** storeDataIdToCid(dataId, cid)5:     dataIdToCid[dataId]←cid6: **end function**7: **function** storeDataIdToHashAndSignature(dataId, hashandsignature)8:     dataIdToHashAndSignature[dataId]←hashandsignature9: **end function**10:**function** storeUserIdToSignature(userId, signature)11:    userIdToSignature[userId]←signature12:**end function**13:**function** getDataCid(dataId)14:    **return** dataIdToCid[dataId]15:**end function**16:**function** getDataHash(dataId)17:    **return** dataIdToHashAndSignature[dataId]18:**end function**19:**function** getUserSignature(userId)20:    **return** userIdToSignature[userId]21:**end function**

#### 3.2.3. Formal Verification of Smart Contracts

In this section, our core discussion focuses on ensuring the accuracy and reliability of smart contract behavior and interactions in the Ethereum blockchain environment. Our smart contracts are designed to manage data content identifiers (CID), data hashes, and access to users’ electronic signatures, covering key operations such as adding data records and querying existing records (See Table 2).

We adopted a methodology based on the Behavior-Interaction-Priority (BIP) framework to expand the formal verification of smart contracts [28]. As a powerful system design tool, the BIP framework allows for the construction of complex system models by integrating behavioral models, interaction patterns, and priority rules, thus providing a solid theoretical foundation for formally verifying smart contracts. In the specific application, we followed the theoretical guidance of T. Abdellatif and K. Brousmiche [29].

(1) Atomic component definition

Each component function of the smart contract is abstracted as an independent ’atomic component’ wherein each component defines a clear set of states and the transition conditions between states. For example, the storeDataIdToCid function links data identifiers with their corresponding CIDs through multiple state phases, such as initialization, processing, and successful or failed storage. Similarly, other functions, such as storeDataIdToHashAndSignature and getUserSignature, follow a similar logic structure, ensuring accurate data storage and retrieval. The relevant function abstraction is shown in Table 2.

(2) Interaction model

The interaction model emphasizes the close relationship between storage and retrieval operations and points out that the normal execution of retrieval functions depends on the success of previous storage steps. In addition, detailed error-handling logic is integrated into the model to ensure that when illegal input or permission problems are encountered, the error situation can be properly fed back to maintain the system’s robustness.

Storage and retrieval: All the retrieval functions (getDataCid, getDataHash getUserSignature) are dependent on the corresponding storage function (storeDataIdToCid storeDataIdToHashAndSignature, storeUserIdToSignature) successfully executed. After successful storage, the relevant data can be retrieved.

Error handling: All storage and retrieval functions include error handling logic to ensure that error states are correctly fed back when input is invalid or access is insufficient.

(3) Verify execution

The contract behavior is simulated and verified by constructing exact properties and logical expressions. We establish four categories of key properties that comprehensively cover functional verification of smart contracts, error-coping mechanisms, idempotence guarantees, and system resilience after transaction failure. This includes but is not limited to verifying the consistency of retrieval results after storage, avoiding misinformation in the event of storage failure, ensuring the stability of repeated storage operations, and system resilience in the face of transaction anomalies.

Defining concrete properties and logical expressions, we have defined four categories of properties that express aspects ranging from basic functional guarantees to error handling and system recovery:

(1) Store and Retrieve consistency: Ensure that every store operation followed by a search operation returns the correct stored value.

Attribute A1: After each successful execution of storeFileIdToCid (dataId, cid), the call to getDataCid (dataId) returns the same cid immediately.

Attribute A2: For every successful execution, storeDataIdToHashAndSignature (dataId hashAndSignature), call getDataHash (dataId) of the same hashAndSignature return immediately.

Attribute A3: After each successful execution of storeUserIdToSignature (userId, signature), the call to getUserSignature (userId) returns the same signature immediately.

(2) Storage failure Handling: This ensures that no error or inconsistent data is returned in the event of a storage failure.

Attribute A4: If the storeFileIdToCid operation fails, subsequent getDataCid calls do not return the previous cid.

(3) Idempotence of store operations: Determining that repeated operations do not result in errors or state anomalies.

Attribute A5: The storeFileIdToCid operation on the same dataId keeps the result consistent even if repeated.

(4) Transaction failure Resilience: Ensures that the system can handle it correctly and recover consistently if an operation fails.

Attribute A6: If the storeFileIdToCid or storeDataIdToHashAndSignature operation fails for any reason, the effective operating system does not collapse or prevent further.

After a strict verification process and attribute inspection, the smart contract performs up to the standard on all the preset attributes A1 to A6. This means that the contract not only ensures the consistent matching between storage and retrieval operations but also properly handles the storage failure, effectively realizes the idempotence operation, and maintains the continuous operation and stability of the system when the transaction is frustrated, which indicates the reliability and security of the contract. The pseudocode of the relevant algorithm is shown in Algorithm 4.
**Algorithm 4** Formal Verification of Smart Contract1:**enum** State {IDLE, STORING, RETRIEVING, SUCCESS, FAILURE}2:**function** performAction(actionType, key, value)3:    switch (actionType)4:    case STORE_CID:5:    **if** currentState == IDLE **then**6:        currentState = STORING7:        storedData[key] = value8:        **if** storedData[key] == value **then**9:           currentState = SUCCESS10:       **else**11:          currentState = FAILURE12:       **end if**13:   **end if**14:   ……15:   case RETRIEVE:16:   **if** currentState == SUCCESS **then**17:       currentState = RETRIEVING18:       **if** storedData.contains(key) and storedData[key] == value **then**19:       **else**20:          currentState = FAILURE21:       **end if**22:   **end if**23:   currentState = IDLE24:**end function**

### 3.3. IPFS Integration with ROS

When evaluating the cost-effectiveness of blockchain technology at the storage level, particularly for large-scale data and digital content storage, we introduced the Interplanetary File System (IPFS) as a more efficient storage solution. IPFS is a distributed storage protocol designed to maximize data storage efficiency and minimize file storage redundancy. When integrated with ROS features, the system enables ROS nodes to utilize the decentralization offered by IPFS. This allows these nodes to share, store, and retrieve files independently of a central storage server. Consequently, each ROS node becomes not only an agent executing its assigned tasks but also an IPFS node responsible for storing and providing data services. This integrated architecture offers several advantages: (a) IPFS significantly reduces data storage space by storing only one unique instance of a file with identical content; (b) designed for long-term storage, the IPFS protocol mitigates issues related to single points of failure and temporary data loss, thereby enhancing the robustness of robotic systems; (c) for frequently requested data, IPFS improves local data accessibility by caching requests along previous paths, substantially lowering data retrieval latency and accelerating response times of robotic systems. Therefore, IPFS has been demonstrated to be more suitable as a storage platform for data compared to traditional servers. Within this system, ROS nodes collaborate as part of the IPFS network, ensuring efficient data sharing and access. The ROS nodes not only collaborate to execute robotic tasks but also provide mutual support in terms of data storage and retrieval. This facilitates data transfer through peer-to-peer networking protocols, thereby significantly improving the overall system’s flexibility, scalability, and decentralization. The relevant algorithms are described in detail in Algorithm 5.
**Algorithm 5** Receive and Publish Data to IPFS1:**procedure**ReceiveAndPublishData2:    *sock* ← Socket(AF_INET, SOCK_STREAM)3:    Bind(*sock*, (*server_ip*, *server_port*))4:    Listen(*sock*, 1)5:    Print‘‘Waitingfordataupload…’’6:    **while true do**7:        *connection*, *address* ← Accept(*sock*)8:        *data_name* ← Receive(*connection*)9:        **if** notdata_name **then**10:          **break**11:       **end if**12:       Print “Received data name: ‘*data_name*’”13:       *save_path* ← JoinPath(*save_directory*, *data_name*)14:       *data* ← Open(*save_path*, ‘*wb*’))15:       **while true do**16:          *data* ← Reveive(*connection*)17:          **if** notdata **then**18:              **break**19:          **end if**20:          Write(*data*)21:       **end while**22:       Close(*data*)23:       $\mathrm{Print}\;‘‘\mathrm{Data}‘\mathrm{data}\_{\mathrm{name}}^{\prime }\;\mathrm{received}\;\mathrm{successfully}’’$24:       Close(*connection*)25:       *data* ← Open(*data_name*, ‘rb’)26:       *response* ← PostToIPFS(*data*)27:       **if** *response*.status_code = 200 **then**28:          cid←response.json()[‘Hash’]29:          PublishCID(cid)30:          Print‘‘CIDuploadedsuccessfully!’’31:       **else**32:          Print‘‘Failedtouploaddata.’’33:       **end if**34:   **end while**35:**end procedure**

In a ROS system, we first store the encrypted data collected from vision sensors in the local data system at the ROS node. Then, employing socket programming, we acquire the IP address and port number of the IPFS node to establish a TCP connection between the ROS node and the IPFS node. Once the encrypted data is accessed, it is divided into appropriately sized chunks to ensure successful transmission over the TCP connection. These data chunks are then sequentially transmitted to the IPFS node using the TCP connection and the socket’s send data API. After each data chunk is received by the IPFS node and its data integrity is confirmed, these chunks are integrated into the IPFS network through API calls provided by IPFS software (version 0.14.0). This process continues until all data blocks are sent, received, and stored within the IPFS network. Subsequently, the TCP connection between the ROS endpoint and the IPFS node is terminated to free resources. In this manner, the encrypted data collected from vision sensors is successfully uploaded to the IPFS network and assigned its unique data hash value (CID), facilitating subsequent data access and sharing.

## 4. Results

We assess the practicality of the system through three key metrics: the rate of message sending and receiving in RabbitMQ, the efficiency and stability of HybridABEnc encryption and decryption processes, and a comparative analysis with the AuthROS system based on experimental data. The software information for the experimental simulation test is detailed in Table 3.

### 4.1. Comparison of Functional Features

We carefully analyze and compare the functional characteristics of many related privacy-sensitive data storage systems in recent years and show our analysis results in detail in Table 4. It is evident from these comparative data that our system demonstrates significant advantages in multiple key functional features. This not only proves the efficiency and advancement of our system but also highlights its uniqueness in dealing with privacy-sensitive information.

### 4.2. RabbitMQ Message Sending and Receiving Rate

The RabbitMQ experiments focus on the rates at which messages are sent and received. To make the experimental results more adaptable, we wrote a Python program to simulate the process of sending and receiving messages. The sender dispatches 10,000 messages to RabbitMQ because RabbitMQ addresses the issue of unsynchronized production and consumption rates through asynchronous calls. Therefore, after sending, the Python program that receives messages is run to test the rate at which messages are received. The results obtained from the test and analysis of these 10,000 sets of data are displayed in Table 5.

In our performance test, the producer node sent 10,000 messages to the RabbitMQ message queue, completing the task in 1.105 s. This demonstrates RabbitMQ’s capacity to push messages at an exceptionally high rate on the producer side, with an average send rate of 9049.77 messages per second. This outcome indicates that RabbitMQ producer nodes are capable of pushing a significant volume of messages to queues within a short timeframe, suggesting that the system maintains robust performance under high-load conditions.

During the test, we simulated three consumer nodes (Consumer 1, Consumer 2, and Consumer 3) at the blockchain end to handle the message processing tasks. Throughout the test period, the three consumer nodes processed 3750, 3750, and 2500 messages, respectively, with corresponding processing times of 4.825, 4.461, and 4.675 s. Analysis of the test data reveals average message processing rates for Consumer 1, Consumer 2, and Consumer 3 of 777.20 messages/s, 840.62 messages/s, and 534.76 messages/s, respectively. Collectively, the three consumer nodes processed 10,000 messages within a span of 4.825 s, achieving an overall processing rate of 2072.54 messages per second.

In RabbitMQ usage scenarios, the high send rate of the producer stands out as a significant advantage, enabling the system to receive and queue tasks for processing large-scale data swiftly. Despite slight variations in processing rates among consumer nodes, the overall level is maintained at a balanced level, ensuring smooth and efficient message handling. A detailed analysis of these test data illustrates that our system, which integrates RabbitMQ, exhibits high throughput and stability in message processing, particularly in high-concurrency scenarios. This confirms the system’s suitability for high-concurrency messaging situations. Furthermore, RabbitMQ’s stability and scalability provide a mechanism for our system to adjust resources dynamically in response to fluctuations in message traffic. For instance, additional consumer nodes can be deployed as necessary to accommodate an increased message load, or cluster configuration can be utilized to enhance redundancy and fault tolerance.

### 4.3. HybridABEnc Algorithm’s Encryption and Decryption Efficiency and Stability

In this paper, we evaluate the performance of the HybridABEnc algorithm as the fundamental privacy protection mechanism within our system. In the experiments, we utilized a dataset consisting of image and video files, totaling 656 files, with the average length of each video ranging between 6 and 7 min [33,34]. To facilitate testing, we use the same attribute list and access policy for the data to perform experimental testing of encryption and decryption. The time complexity of the encryption and decryption algorithm can be expressed as O(n · f(m)) and O(n · g(m)), where n represents the number of files to be processed and m represents the average data size of the file. In the representation, f(m) and g(m) represent the time complexity required to perform encryption and decryption operations on a single file, respectively. The specific form of these two functions depends on a number of factors, including the detailed implementation of the encryption and decryption algorithm, the size of the data attribute set, the complexity of the access control policy, and the size of the file data itself. The experimental results indicate that the HybridABEnc algorithm achieved a robust encryption rate of 61.70 MB/s in our setup, ensuring the system can uphold an efficient data processing workflow while safeguarding privacy. This underscores its capability to manage substantial data volumes efficiently. Furthermore, we observed an overall encryption latency of 30.11 s, underscoring the algorithm’s ability to expedite the encryption process and thereby providing substantial evidence of the system’s swift response capability. The study also delves into the decryption performance. The decryption throughput recorded was 38.63 MB/s, which while lower than the encryption rate, remains adequate for the needs of typical application scenarios. Moreover, the average decryption time was remarkably short at 0.0733 s, providing users with nearly instantaneous access to the decrypted data. This emphasizes the system’s efficiency and quick data retrieval capability. The pertinent experimental results are compiled in Table 6.

In the work of Hujare R et al., file storage security relies on the integrity of Solidity smart contracts, the persistence and immutability of the blockchain, etc. However, no specific encryption scheme is provided to ensure file security [35]. While our system uses IPFS, blockchain, and other technologies to guarantee the security of system permissions, the HybridABEnc encryption scheme guarantees the security of the data itself, adding another layer of security. In the study by L. Li et al., the traditional AES+RSA encryption scheme offers basic security and demonstrates specific performance metrics [36]. For example, the average times for the AES algorithm to encrypt and decrypt fire IoT data are 24.98 ms and 24.78 ms, respectively. The average time for RSA to generate user accounts is 21.22 ms. However, our HybridABEnc solution offers more significant advantages across various measurement dimensions. The HybridABEnc encryption scheme achieved a throughput of 61.70 MB/s, significantly surpassing the performance of AES + RSA, an important metric for processing large data volumes and enabling efficient real-time data streaming. Although the overall latency for encryption is 30.11 s, this is acceptable for non-real-time data processing. It is especially beneficial given its additional privacy protection and access control advantages.

Furthermore, our solution offers a more advanced and flexible approach than RSA for private data sharing and role definition by establishing access control policies based on user attributes. This enhanced level of access control renders the system more suitable for highly customized data rights management needs. In terms of decryption performance, although the decryption throughput (38.63 MB/s) is lower than the encryption rate, it is acceptable because the access policy used for decryption is complex in the list of attributes used for encryption. At the same time, our solution still provides a very low average decryption latency (0.0733 s), and the decryption rate is lower than the encryption rate. Users can access and process the decrypted data almost instantly, further validating the utility of our solution.

### 4.4. Performance Comparison of PrivShieldROS on ROS Nodes and AuthROS

It can be seen from the experimental data that in the face of large-scale and complex data type transmission tasks, our system adopts a security scheme based on hybrid attribute encryption (HybridABEnc), which can effectively ensure security and show extremely high encryption efficiency. Our system effectively utilizes the stability and reliability of the TCP protocol to perform large-scale and complex data transmission tasks. To verify the performance and efficiency of the system, we conducted a comprehensive test using a dataset of 10,000 images (totaling 1,274,678,532 bytes) as a test object. Through careful measurement and analysis, we found that the average time of the encryption process reached 0.0146 s, the longest time was only 0.0496 s, and the shortest time was 0.0090 s. This result accurately reflects the high-performance level of our system in the encryption step and fully proves the encryption efficiency of our scheme. At the same time, the system demonstrated an average encryption rate of 8,730,114.83998 bytes/s. In terms of upload rate, the average upload rate of our system is 1.4112 MB/s, which verifies the efficient data transmission capability at the network level. In contrast, the AuthROS system uses connectionless User Datagram Protocol (UDP) and SM4 symmetric encryption algorithms to process 10,000 messages (i.e., simple string data). Although their test data showed that with SM4 encryption supported, the average response time was 0.852 ms, with peak times between 0.50 ms and 1.23 ms. However, due to the connectionless transmission, the packet loss rate is 0.14%, which leads to shortcomings in data integrity and transmission stability. In particular, the prominent problem of packet loss rate is an inherent drawback of UDP transmission methods, which is undoubtedly a big problem for systems that must ensure data integrity. The relevant experimental data are shown in Table 7.

By comparing our system with the AuthROS system, we can find several key advantages of our system compared with the AuthROS system. First, using TCP rather than UDP is a wise choice for the stable transfer of large data sets. As a reliable transmission control protocol, TCP ensures that data is not lost or corrupted during transmission. Second, the HybridABEnc encryption scheme provides stronger security for data encryption and supports flexible access control mechanisms, which is essential to support multi-user web applications. Third, our system has shown impressive efficiency in performance tests, not only in the encryption rate, which has a prominent performance but also in the upload rate, which shows superior performance at the network level. In addition, our system introduces more novel concepts than the AuthROS system, such as attribute-based encryption (ABE), which allows users to gain access to data based on their attributes rather than just their identity. This encryption mechanism makes data sharing more efficient. It provides a solution for complex rights management needs, thus showing outstanding application potential in areas where security and data personalization are more demanding.

Therefore, the comparison with the AuthROS system has confirmed that our system, which is based on the mixed attribute encryption scheme, performs efficiently and reliably in large-scale and complex data environments. Our system not only improves the security and access control mechanism but also strengthens the integrity and stability of the data transmission process. It has shown better performance when compared to the existing scheme. As the demand for greater security and more efficient data transfer continues to grow, our systems are expected to play an increasingly important role in data-intensive applications.

## 5. Conclusions

In this paper, we propose a privacy-sensitive data storage system for ROS vision sensors, which aims to solve the data security challenges encountered in various application fields with high privacy protection. This system provides an innovative solution for secure storage and reliable sharing in the ROS environment by integrating decentralized blockchain technology and interplanetary file system IPFS. By taking advantage of the immutability of blockchain and the highly distributed storage characteristics of IPFS, we significantly enhance the anti-tampering ability, reliability, and retrieval efficiency of data. In addition, combined with the HybridABEnc solution, KP-ABE’s fine-grained attribute-based access control mechanism for sensitive privacy data and the efficiency of symmetric encryption were integrated, thereby increasing the efficiency and flexibility of data transmission processing.

The design of this system has a wide range of application prospects, especially suitable for scenarios with high privacy protection requirements. For example, in industrial automation, systems can ensure the secure storage of sensitive information during production and support efficient data sharing and processing. In autonomous transportation and smart city applications, the system can realize the protection of location data and user personal information and ensure real-time information circulation. The system can record and store medical data and patient information in the healthcare domain to ensure privacy protection while supporting efficient data access. These potential practical applications highlight our system’s broad applicability and effectiveness for processing and protecting vision sensor data.

Although the system has demonstrated significant advantages, its practical deployment and application process may face several challenges. Firstly, technical compatibility issues may become a major obstacle, especially the complexity of integrating the existing ROS environment with this system. At the same time, resource constraints, including the limitations of computing resources and storage capacity, may affect system performance, especially in resource-constrained environments. In addition, user acceptance is also a factor that cannot be ignored. The successful implementation of the system depends on the user’s acceptance and trust in the new technology.

In response to the above challenges, our future research will be devoted to further optimizing the design and implementation of the system. Specifically, the research will focus on enhancing the compatibility and performance of the system and reducing the requirements of computing and storage resources through algorithm optimization to adapt the system to a wider range of application scenarios. At the same time, we will also work to improve the system interface and user experience to promote user recognition and adoption of the technology. In addition, with the improvement of computing power and the evolution of attack methods, further enhancing the security of existing encryption schemes is necessary. Therefore, we will explore the potential integration of advanced encryption techniques, not limited to systems with integrated attribute-based encryption schemes, to enhance data security further.

Therefore, we considered the following directions to enhance the data protection mechanism of the existing system:

(1) Integration of homomorphic encryption techniques: Considering the power of homomorphic encryption, it is possible to operate on encrypted data without decryption, which is particularly important for protecting user privacy. In future work, we will consider evaluating how homomorphic encryption works with the current HybridABEnc scheme, especially in guaranteeing efficient data processing.
(2) Add zero-knowledge proofs: Zero-knowledge proofs provide a mechanism for one party to prove to another that it has certain information without revealing it. In future applications, we would like to consider using this technique to enhance user authentication and data access control without revealing unnecessary personal or sensitive information.

While exploring these advanced encryption technologies, we are also aware of the challenges in the integration process, including algorithm performance, system compatibility, and user acceptance. Therefore, our future research will not be limited to the selection and experimental validation of technologies but will also include how efficiently these technologies can be integrated into existing architectures and the evaluation of the combined impact on system performance, security, and user experience.

Therefore, the data storage system based on blockchain and IPFS technology proposed in this study provides an innovative solution for secure storage, real-time sharing, and privacy protection of visual sensor data in the ROS environment. Implementing this system can significantly enhance the protection ability of existing application scenarios for sensitive visual data and improve the flexibility and efficiency of data processing and application. Future optimization and research will further improve the adaptability and practicality of this system in different fields and provide solid technical support for robotics applications requiring high privacy protection. In addition, in order to ensure the practicality and wide applicability of this system, we will consider cooperating with industry partners in the future to actually deploy and verify the system in various robotic applications, providing solid technical support for robotic application fields requiring high privacy protection through continuous research and cooperation, and further optimizing the system to adapt to different usage scenarios. While contributing to data security and privacy protection in the future ROS environment, this work also has potential value in other fields such as cloud or edge robotics [37,38,39,40,41].

## Figures and Tables

**Table 1:** Smart contract gas consumption.

Name	Index	Value
Smart contract deployment	Gas used	735,980 (0xb3aec)
storeDataIdToCid function	Gas used	84,297
storeDataIdToHashAndSignature function	Gas used	434,466
storeUserIdToSignature function	Gas used	95,174

**Table 2:** Smart contract function abstraction.

	Feature	State	Conversion
storeDataIdToCid	Associate the data ID with the corresponding CID store	Init, Storing, Success, Fail	Init to Storing: Starts processing the storage request. Storing to Success: CID storing to success. Storing to Fail: Storing to fail.
storeDataId To-HashAndSignature	Associate data ID with their corresponding hash and signature stores	Init, Storing, Success, Fail	Init to Storing: Starts processing the storage request. Storing to Success: The hash and signature are successfully stored. Storing to Fail: Storing to fail.
storeUserIdToSignature	Associate the user ID with the corresponding signature store	Init, Storing, Success, Fail	Init to Storing: Starts processing the storage request. Storing to Success: The signature is successfully stored. Storing to Fail: Storing to fail.
getDataCid	Retrieve the corresponding CID based on the data ID	Init, Fetching, Retrieved, NotFound	Init to Fetching: This starts the retrieval request. Fetching to Retrieved: The CID is successfully retrieved. Fetching to NotFound: The CID is not found.
getDataHash	Retrieve the corresponding hash and signature based on the data ID	Init, Fetching, Retrieved, NotFound	Init to Fetching: This starts the retrieval request. Fetching to Retrieved: The hash and signature are successfully retrieved. Fetching to NotFound: The hash and signature are not found.
getUserSignature	Retrieve the corresponding signature based on the user ID	Init, Fetching, Retrieved, NotFound	Init to Fetching: This starts the retrieval request. Fetching to Retrieved: The signature is successfully retrieved. Fetching to NotFound: The signature is not found.

**Table 3:** Configuration information.

Name	Version
CPU	11th Gen Intel(R) Core(TM) i7-11800H @ 2.30 GHz (Intel, Santa Clara, CA, USA)
Memory	16.0 GB
Hard disk	NVMe SAMSUNG MZVL2512HCJQ-00BH1
System environment	Ubuntu22.04
Programing language	Python3.7, 3.10

**Table 4:** Comparison of co-energy characteristics of different schemes.

Project Proposal	Blockchain-Based (Decentralized)	Based on IPFS	Fine-Grained Access Control	Privacy Protection
ref [1]	✔	✘	✘	✔
ref [30]	✘	✘	✔	✔
ref [31]	✔	✔	✘	✔
ref [32]	✘	✘	✔	✔
Proposed scheme	✔	✔	✔	✔

**Table 5:** rabbitMQ test result.

Characters	Message Total	Total Time (s)	Average Processing Rate (Messages per Second)
producer	10,000	1.105	9049.77
Consumer 1	3750	4.825	777.20
Consumer 2	3750	4.461	840.62
Consumer 3	2500	4.675	534.76
Total	10,000	4.825	2072.54

**Table 6:** Encrypting and decrypting performance data results.

Performance Index	Value
Total number of encrypted and decrypted bytes	1,947,913,076 bytes
Total number of encrypted and decrypted files	656 documents
Encryption throughput	64,692,152.89 bytes/s
Average single file encryption latency	0.0459 s
Total encryption time	30.11 s
Decryption throughput	40,508,382.55 bytes/s
Average single file decryption delay	0.0733 s
Total decryption time	48.09 s

**Table 7:** ROS node data sending experiment results.

Performance Index	Value
Total number of uploaded files	10,000
The total size of all files in the folder	1,274,678,532 bytes
Total encryption time	146.00911 s
Average encryption time	0.0146 s
Maximum encryption time	0.0496 s
Minimum encryption time	0.0090 s
Average encryption rate	8,730,114.83998 bytes/s
Average upload rate	1.4112 mb/s

## Data Availability

The codes and datasets involved in this study are available at https://github.com/qwertynji/PrivShieldROS.git (accessed on 21 March 2024).
